# New Potential Biomarkers for Chronic Kidney Disease Management—A Review of the Literature

**DOI:** 10.3390/ijms22010043

**Published:** 2020-12-22

**Authors:** Irina Lousa, Flávio Reis, Idalina Beirão, Rui Alves, Luís Belo, Alice Santos-Silva

**Affiliations:** 1 UCIBIO\REQUIMTE, Laboratory of Biochemistry, Department of Biological Sciences, Faculty of Pharmacy, University of Porto, 4050-313 Porto, Portugal; irina.filipa@hotmail.com (I.L.); luisbelo@ff.up.pt (L.B.); 2 Institute of Pharmacology & Experimental Therapeutics, & Coimbra Institute for Clinical and Biomedical Research (iCBR), Faculty of Medicine, University of Coimbra, 3000-548 Coimbra, Portugal; freis@fmed.uc.pt; 3 Center for Innovative Biomedicine and Biotechnology (CIBB), University of Coimbra, 3004-504 Coimbra, Portugal; 4 Clinical Academic Center of Coimbra (CACC), 3000-075 Coimbra, Portugal; 5 Universitary Hospital Centre of Porto (CHUP), 4099-001 Porto, Portugal; diretora.depg@chporto.min-saude.pt; 6 Institute of Biomedical Sciences Abel Salazar (ICBAS), University of Porto, 4050-313 Porto, Portugal; 7 Nephrology Department, Coimbra University Hospital Center, 3004-561 Coimbra, Portugal; ruimbalves@hotmail.com; 8 University Clinic of Nephrology, Faculty of Medicine, University of Coimbra, 3000-075 Coimbra, Portugal

**Keywords:** chronic kidney disease (CKD), tubular lesions, endothelial dysfunction, oxidative stress, metabolomic

## Abstract

The prevalence of chronic kidney disease (CKD) is increasing worldwide, and the mortality rate continues to be unacceptably high. The biomarkers currently used in clinical practice are considered relevant when there is already significant renal impairment compromising the early use of potentially successful therapeutic interventions. More sensitive and specific biomarkers to detect CKD earlier on and improve patients’ prognoses are an important unmet medical need. The aim of this review is to summarize the recent literature on new promising early CKD biomarkers of renal function, tubular lesions, endothelial dysfunction and inflammation, and on the auspicious findings from metabolomic studies in this field. Most of the studied biomarkers require further validation in large studies and in a broad range of populations in order to be implemented into routine CKD management. A panel of biomarkers, including earlier biomarkers of renal damage, seems to be a reasonable approach to be applied in clinical practice to allow earlier diagnosis and better disease characterization based on the underlying etiologic process.

## 1. Introduction

Chronic kidney disease (CKD) is a long-term condition characterized by a progressive and irreversibly loss of kidney function or persisting renal damage [1]. CKD has been recognized as a global public health problem. Estimation reports on prevalence, as well as on related morbidity and mortality, confirmed the high socioeconomic burden of this disease [2], particularly due to progression to end-stage renal disease (ESRD) and association with cardiovascular disease (CVD).

**Figure 1:**
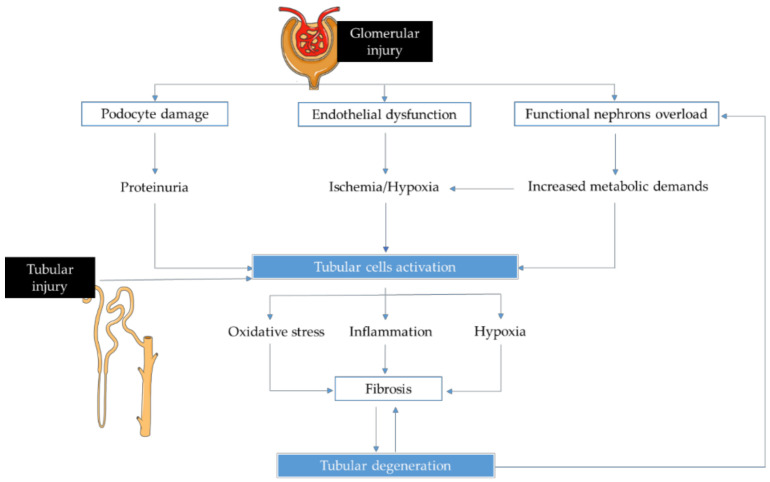
Mechanisms and related consequences of tubular cells activation, triggered by tubular and glomerular injuries.

Irrespective of the etiology of CKD, various structural and functional changes within the kidney will develop during the disease course, resulting in glomerular, tubular and vascular injuries [3]. The progression phase of the disease is characterized by a persistent state of inflammation, hypoxia and oxidative stress that contribute to the development of renal fibrosis [4] (Figure 1).

In clinical practice, glomerular filtration rate (GFR) estimation and albuminuria are widely used for CKD diagnosis and prognosis. GFR estimation correlates with the degree of kidney dysfunction, while albuminuria identifies the presence of renal damage. However, these traditional biomarkers only increase when a significant filtration capacity has been lost and kidney damage is advanced [5]. Thus, they increase when several injuries have already occurred in renal cells [6,7]. Early identification of CKD is an important unmet medical need, not only to predict and prevent CKD progression, but also to further improve patients’ survival and reduce associated morbidities. Hence, more sensitive and early biomarkers are needed to achieve this goal.

Over the last twenty years, several new biomarkers were identified as promising candidates for CKD management. In this review, we selected some of the most well-studied and well-defined biomarkers, which are associated with different pathophysiological mechanisms underlying CKD initiation and progression. We summarize the more important results from published studies on selected biomarkers of renal function, tubular lesions, endothelial dysfunction, and inflammation. A section on novel metabolomic biomarkers is also included due to the increasing attention towards metabolomic analysis in this field. A search on PubMed of human studies published between 2015 and 2020 was conducted using the keywords “CKD”, “chronic kidney disease” and “chronic renal insufficiency”; the biomarker name (s) to search the title or abstract; or the MeSH term “Metabolomics”. From the retrieved articles, and after title and abstract screening, we selected studies that evaluate the validity of the new biomarkers on CKD diagnosis and prognosis in patients from different backgrounds. Additionally, we searched for additional publications in the references of the selected articles.

## 2. New Biomarkers for Chronic Kidney Disease Management

Renal disease is caused by an initial loss of nephrons; then, as a consequence of the kidney functional adaptations to the damage, disease progresses sequentially through different pathophysiological processes, leading to an irreversible state of fibrosis. Moreover, tubulointerstitial hypoxia, inflammation and oxidative stress are simultaneously a cause and effect of renal injury and form a vicious cycle in CKD progression.

**Figure 2:**
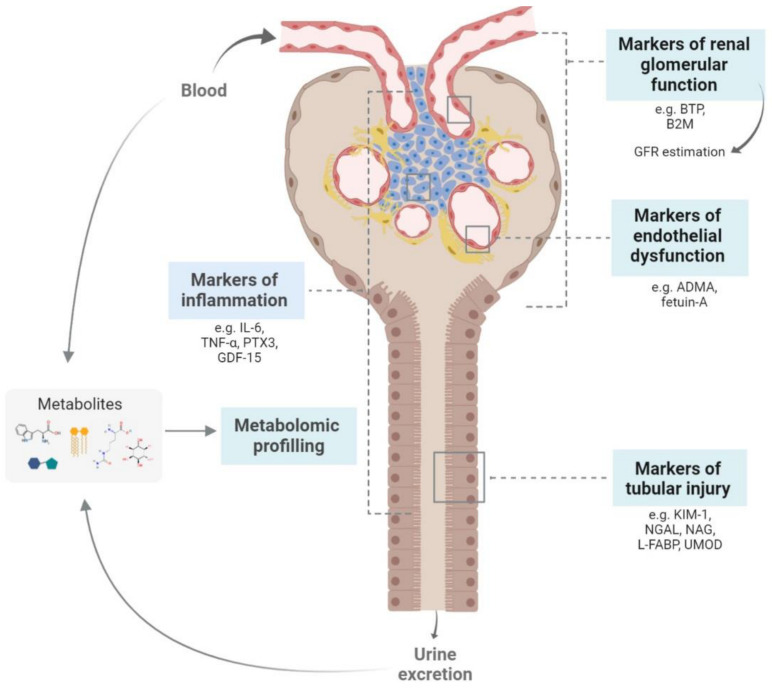
Biomarkers of chronic kidney disease according to the anatomic localization and/or site of production.

The search for new biomarkers should focus on better indicators of renal dysfunction than GFR, and on markers of specific types of kidney injury, assessed in serum and/or urine. The study of specific biomarkers would allow the identification of kidney damage, and they should reflect the underlying pathophysiological processes of kidney damage [8], namely, changes in renal function, tubulointerstitial damage, endothelial dysfunction and inflammation, and/or cardiovascular risk (Figure 2).

### 2.1. Biomarkers of Renal Function

#### 2.1.1. Beta Trace Protein (BTP) and β2-Microglobulin (B2M)

The assessment of renal function relies mainly on the estimation of GFR, using creatinine or creatinine and cystatin C-based equations. Because of the known limitations of these markers, several alternative markers have been studied, namely beta trace protein (BTP) and β2-microglobulin (B2M) (Table 1).

Beta trace protein and β2-microglobulin are low molecular weight proteins that are filtered by the glomeruli and almost completely reabsorbed by the proximal tubules. Since its urinary excretion is residual, the increase in these proteins has been proposed as potential serum markers of decreased GFR, and also as markers of tubular damage if its urinary excretion is elevated [9]. According to Inker et al. (2016) and Foster et al. (2017), BTP and B2M are not affected by ethnicity, and less influenced by age and gender than serum creatinine [10,11].

BTP, also known as prostaglandin D2 synthase, catalyzes the conversion of prostaglandin H2 to prostaglandin D2. It has been used as a marker of cerebrospinal fluid leakage since it is an important constituent of cerebral spinal fluid and is in much lower concentrations in blood [12]. Many studies have described and compared the diagnostic performance of BTP with the traditional markers of CKD [13,14,15], reporting that increased urinary and systemic BTP concentrations were highly correlated with creatinine and cystatin C concentrations [16,17]; moreover, the urinary concentration of BTP progressively increased, along with GFR reduction, and with a rise in its serum concentration [16]. A study conducted by Motawi et al. (2017) showed that serum BTP was higher in type 2 diabetes (T2D) CKD patients with microalbuminuria than in patients with normoalbuminuria, suggesting the potential of BTP in the early diagnosis of diabetic nephropathy [18]. These results are in accordance with previous findings from Dajak et al. (2011) showing high diagnostic accuracy for BTP to detect albuminuria (>30 mg/g) [19].

B2M is a component of the major histocompatibility class I molecule family that is expressed on the surface of most nucleated cells and is present in most biological fluids [20]. Serum and urinary concentrations of B2M increase with the progression of CKD and are particularly high in ESRD patients [20,21].

Several studies have also documented the association of both BTP and B2M with progression to ESRD, CVD development, and mortality [22,23,24]. In spite of the association of BTP and B2M with ESRD, only B2M levels were associated with mortality in a 14-year follow-up study in 250 T2D Pima Indians [22]. However, a larger study, comprising 3613 adults from the Chronic Renal Insufficiency Cohort study, showed that both BTP and B2M were independent predictors of ESRD and all-cause mortality. A cohort study carried out in 9703 participants from the Atherosclerosis Risk in Communities study confirmed the potential utility of measuring changes in B2M to predict worsening to ESRD. A 30% decline in kidney function, assessed using this novel filtration marker, was strongly associated with ESRD [24].

Some researchers used BTP and B2M to develop newer estimated GFR (eGFR) equations. However, in most studies, no advantages were found over the traditional equations used in traditional practice. Pottel et al. (2018) and White et al. (2019) described no additional benefits of adding BTP and B2M to creatinine and cystatin C equations [25,26]. However, a meta-analysis that included 23,318 individuals from six different studies compared the improvements in reclassification of eGFR, risk prediction of ESRD and mortality using BTP- and B2M-based eGFR equations. Compared to the traditional creatinine-based equations, eGFR BTP- and B2M-based equations improved the risk associations and fairly improved the prediction for ESRD and mortality (after adjustment for established risk factors). Moreover, the best performance was found using an eGFR equation that included a combination of both markers [27].

#### 2.1.2. Klotho

Klotho is a transmembrane protein, mainly expressed in the proximal and distal tubular cells [28]. CKD patients present reduced levels of klotho. Klotho deficiency is positively correlated with kidney function decline, even in CKD patients at stages 1 and 2 [29,30]. Therefore, klotho was previously described as an extremely sensitive and early marker in CKD [29] since its levels reflect the degree of renal insufficiency. In accordance with such findings, Qian et al. (2018) reported that changes in soluble klotho could be used as indicators of CKD progression, since they correlated with changes in eGFR [31]. Likewise, a meta-analysis by Wang et al. (2018) reported a significant positive correlation between soluble klotho levels and eGFR in CKD patients [32].

In CKD patients, decreased klotho levels were also associated with increased albumin excretion [33], higher risk of CVD [34], mortality [35], and CKD related inflammation [36]. Moreover, klotho has a pathophysiological role in ion disorders, and might be used as a marker of abnormal phosphate and bone metabolism in these patients. Zheng et al. (2018) demonstrated that serum klotho levels were associated with the degree of mineral bone disorder in a group of 125 hemodialyzed patients. Patients with osteoporosis presented lower klotho levels than those with normal bone mass or with osteopenia [37]. In addition, a study conducted on 152 CKD patients showed that klotho levels were negatively correlated with serum phosphate levels, suggesting that decreased klotho aggravates the urinary phosphate excretion disorder [38].

### 2.2. Biomarkers of Tubular Lesions

Tubular function is impaired early in the disease course—sometimes, even before evident glomerular dysfunction. Several promising new biomarkers of tubular integrity have been studied (Table 2), namely neutrophil gelatinase-associated lipocalin (NGAL), kidney injury molecule-1 (KIM-1), *N*-acetyl-β-D-glucosaminidase (NAG), liver-type fatty acid binding protein (L-FABP), and uromodulin (UMOD).

#### 2.2.1. Neutrophil Gelatinase-Associated Lipocalin (NGAL), Kidney Injury Molecule-1 (KIM-1) and *N*-acetyl-β-D-glucosaminidase (NAG)

The importance of neutrophil gelatinase-associated lipocalin (NGAL), kidney injury molecule-1 (KIM-1) and *N*-acetyl-β-D-glucosaminidase (NAG) as early indicators of tubular lesions has been documented, but some conflicting results were found. NGAL and KIM-1 are expressed in tubular epithelial cells in response to injury and have been proposed as early biomarkers of CKD [47,48]; their values are increase before the development of irreversible tubular atrophy and fibrosis.

NGAL is a ubiquitous lipocalin iron-carrying protein, mainly secreted by activated neutrophils in response to bacterial infection. It is also expressed in the renal tubular epithelial cells and released in case of cell damage [47]. Both systemic and urinary NGAL concentrations increase when tubular kidney damage occurs [49]. It is believed that NGAL has a protective role in case of renal damage, since it contributes to the recovery of the epithelium after injury [50]; it appears as a biomarker of tubular renal injury, and not of renal (dys)function. NGAL appears as a promising biomarker for early stages of CKD, since it has been associated with early decline in eGFR and albuminuria [49]. The findings of Abbasi et al. (2020) suggest that urinary NGAL levels increase before the onset of traditional markers (eGFR and albuminuria) in T2D patients [51], which indicates that urinary NGAL could be a complementary measurement in the early diagnosis of diabetic nephropathy. In line with this, a recent meta-analysis conducted by Kapoula et al. (2019) aimed to evaluate the diagnostic accuracy of NGAL for early predicting diabetic nephropathy. This analysis enrolled 22 studies, comprising a total of 683 healthy individuals and 3249 diabetic patients (488 with type 1 diabetes (T1D) and 2761 with T2D). The authors found that both serum and urinary NGAL showed an increasing trend alongside with albuminuria and eGFR aggravation. The highest concentrations of NGAL were found in patients with the highest disease severity. Moreover, NGAL showed a moderate to high capacity for early detection of diabetic nephropathy. Most previous published studies reported a similar tendency regarding NGAL for the diagnosis of diabetic nephropathy [52].

KIM-1 is a cellular receptor responsible for regulating immune cells activity in response to viral infections. It is not detectable in the normal kidney, but elevated levels were found in experimental and clinical kidney damage [48]. KIM-1 is a recognized biomarker for acute kidney injury (AKI), and it is also upregulated in CKD [53]. Zhang et al. (2018) reported that higher baseline concentrations of KIM-1 were associated with higher odds of incident CKD in a cohort of 324 adults with hypertension but without baseline kidney disease [54]. In patients with T2D and early stages of CKD (G1 and G2), serum and urinary NGAL, as well as urinary KIM-1, were increased in association with increased urine albumin-to-creatinine ratio, and appeared as significant predictors of albuminuria [55]. In a follow-up study of 527 adults with T1D, Bjornstad et al. (2018) reported that only NGAL (and not KIM-1) was associated with urine albumin-to-creatinine ratio and incident impaired GFR [56].

NAG is a glycosidase found mainly in the lysosomes of proximal tubular cells. Due to its molecular weight (130,000 Dalton), NAG cannot be filtered at the glomerulus; thus, increased urinary concentrations of NAG are signs of proximal tubular damage [57].

In order to predict progression of CKD in patients with chronic heart failure, Jungbauer et al. (2016) evaluated urinary NAG, NGAL and KIM-1 concentrations in 149 patients, who were followed for a period of 5 years. Strong associations of CKD progression with NAG and KIM-1, but not with NGAL, were found in these patients [58], suggesting their usefulness as cardiorenal markers. However, in a prospective cohort study comprising 250 patients at all CKD stages, NGAL was more strongly correlated with disease progression in more advanced CKD stages than KIM-1 and NAG. In addition, NGAL was the only predictor of ESRD and death [59].

The conflicting results may suggest that NGAL, KIM-1 and NAG have different behaviors depending on the CKD cause. Further studies should focus on the kinetics of NGAL, KIM-1 and NAG in CKD with different etiologies to identify their specific field as renal disease biomarkers.

#### 2.2.2. Liver-Type Fatty Acid Binding Protein (L-FABP)

The liver-type fatty acid binding protein (L-FABP) is abundantly expressed in hepatocytes and in proximal renal tubular cells. Injury of the proximal tubular cells induces upregulation of the L-FABP gene, leading to increased L-FABP expression by these cells and, consequently, to an increase in the urinary L-FABP excretion [60]. The urinary levels of L-FABP have been correlated with the degree of tubulointerstitial damage in renal biopsies [61].

Urinary L-FABP seems to be useful in predicting AKI [62] and also AKI-to-CKD transition [63]. In T2D patients, L-FABP appears to be a more sensitive marker than proteinuria to predict CKD progression. Accordingly, Kathir et al. (2017) reported that urinary L-FABP was associated with a decline in GFR in CKD patients without albuminuria [64]. A prospective, observational, multicenter study, comprising 244 Japanese patients with CKD, correlated higher urinary L-FABP levels with the development of ESRD and CVD, irrespective of diabetes. Non-fatal or fatal CVD events and progression to ESRD were associated with higher L-FABP levels and low eGFR [65]. Similarly, Maeda et al. (2015) showed that L-FABP, as well as urinary albumin-to-creatinine ratio, could be useful in assessing cardiovascular damage in T2D patients at CKD stages 1 and 2, since they correlated with the elevation of cardiac markers and electrocardiogram abnormalities [66].

#### 2.2.3. Uromodulin (UMOD)

Uromodulin (UMOD), also known as Tamm–Horsfall protein, is a kidney-specific protein, exclusively produced by the renal tubules, and it is involved in the control of water–electrolyte balance and in the defense against urinary tract bacterial infections [67]. Under normal conditions, UMOD is the most abundant protein in urine. In the case of CKD patients, with tubular atrophy and interstitial fibrosis, the urinary and serum UMOD concentrations are reduced. Rare mutations in the UMOD gene were associated with hereditary autosomal-dominant tubulointerstitial diseases [67].

An observational study that included 170 CKD patients at stages 1 to 5 pre-dialysis and 30 healthy individuals showed that serum UMOD levels were significantly lower in CKD patients, and correlated negatively with serum creatinine and cystatin C levels and positively with eGFR. Moreover, UMOD circulating levels were highly accurate in the assessment of CKD stages, showing a gradual decline with the progression of kidney disease [68]. Accordingly, Steubl et al. (2016) showed that plasma UMOD was capable of differentiating patients without CKD and with stage 1 CKD, contrarily to creatinine and cystatin C [69]. Furthermore, Scherberich et al. (2018) reported that declining serum UMOD concentrations are associated with loss of kidney function in patients with CKD at stages 1 to 5 and autoimmune kidney diseases, even in the absence of creatinine changes. Decreased levels of serum UMOD were further associated with the structural integrity of the renal parenchyma [70].

### 2.3. Biomarkers of Endothelial Dysfunction

Endothelial dysfunction begins in the early stages of CKD and progresses with renal disease severity [83,84,85,86], suggesting that it may be the key element linking CKD to the increased CV risk in these patients. The mechanisms underlying endothelial dysfunction in CKD include reduced nitric oxide (NO) production due to increased levels of endogenous NO synthase inhibitors, oxidative stress, inflammation, and vascular calcification [87,88]. Several endothelial dysfunction markers have been studied in the context of CKD. In this study, we focused on asymmetric dimethylarginine (ADMA) and fetuin-A, since these biomarkers reflect distinct pathophysiological alterations affecting endothelium integrity (Table 3).

#### 2.3.1. Asymmetric Dimethylarginine (ADMA)

Asymmetric dimethylarginine (ADMA) is the most effective endogenous inhibitor of NO synthase, and its accumulation contributes to endothelial dysfunction and, therefore, to atherosclerotic changes [89]. In fact, ADMA is an independent risk marker for CVD development and for all-cause mortality [90].

In a recently published study, ADMA was positively associated with endothelial dysfunction and with CKD duration and severity in pre-dialysis patients [86]. Other studies found similar associations between ADMA levels and eGFR. A cross-sectional study conducted on 176 CKD patients showed a correlation between increasing plasma levels of ADMA and kidney function deterioration; moreover, stage 5 patients registered the highest elevation on ADMA plasma levels. The increase in ADMA was associated with by both eGFR < 60 mL/min/1.73 m^2^ and anemia, two risk factors for CVD [84]. Inverse associations between eGFR and ADMA concentrations were also found in an elderly Korean cohort; in this study, the mean ADMA levels were significantly higher in subjects with eGFR < 60 mL/min/1.73 m^2^ than in those with a higher eGFR [91]. A systematic review and meta-analysis conducted by Wang et al. (2018) that analyzed six prospective and cross-sectional studies correlated circulating ADMA concentrations with carotid intima-media thickness in CKD patients. Since carotid intima-media thickness is an important predictor of atherosclerosis, the researchers proposed that elevated circulating ADMA levels are predictors of atherosclerotic disease development in these patients, and not only a marker of subclinical atherosclerosis [92]. Furthermore, Bartnicki et al. (2016) found that left ventricular structural and functional pathological changes occur in parallel with elevated serum ADMA concentrations [93]. Triches et al. (2018) showed that higher circulating ADMA levels were associated with new-onset microalbuminuria and/or with a progression in initial albuminuria in at least 30% in a follow-up study of hypertensive and both hypertensive and diabetic patients [85]. An increase in ADMA may precede albuminuria development, suggesting that ADMA levels are an earlier biomarker of CKD in this population. Subjects who presented increased ADMA values during the follow-up period progressed to the later stages of CKD [85]. Moreover, the magnitude of the ADMA increase might be a marker of the rate of kidney disease progression. Additionally, Seliger et al. (2016) demonstrated in a group of older hypertensive patients with and without CKD, that laser Doppler flowmetry-based measures of endothelial dysfunction and microvascular reactivity are the strongest determinants of albuminuria, irrespective of diabetes status [94]. This study provides further evidence that endothelial function is correlated with albuminuria and that albuminuria may reflect glomerular microvascular dysfunction.

#### 2.3.2. Fetuin-A

Fetuin-A, a vascular calcification inhibitor, is also a risk factor for the development of endothelial dysfunction in CKD patients [95]. Reduced serum levels of fetuin-A have been associated with increased CV mortality in maintenance hemodialysis patients [96]. However, the relationship between vascular calcification and endothelial dysfunction is not so clear in patients with early CKD.

Mutluay et al. (2019) conducted a study on 238 CKD patients at stages 3 and 4 and with ESRD in order to compare fetuin-A levels between them. Fetuin-A levels were significantly lower in ESRD patients than in patients with stages 3 or 4 of CKD. Moreover, in ESRD patients, the lower levels of fetuin-A were associated with higher vascular calcification scores and carotid intima-media thickness [97]. A meta-analysis of 13 studies comprising 5169 patients at all stages of CKD aimed to determine the relationship between circulating fetuin-A levels and the risk for all-cause mortality. Data collected for this meta-analysis by Zhou et al. (2019) suggest that there is a significant association between low fetuin-A levels and higher risk of mortality, independent of diabetes and inflammation, in dialysis patients, but not in non-dialysis patients. In dialyzed patients, a 0.1 g/L increase in fetuin-A levels lowers the risk for all-cause mortality in 8% [98].

In addition to its role in endothelial dysfunction, fetuin-A has also been associated with inflammation [96,99] and nutritional status [100,101], which is a common complication associated with increased mortality in CKD patients [102]. Dialyzed patients have poorer nutritional status than non-dialyzed patients [103], which might explain the differences in fetuin-A levels found between these populations.

### 2.4. Biomarkers of Inflammation

Several studies in the literature suggest that activation of the inflammatory processes in the early stages of CKD drives kidney function impairment and propose that the assessment of inflammatory markers might help in earlier CKD diagnosis. Increased inflammatory markers and changes in GFR have been widely reported [108,109,110]. Moreover, inflammation is a risk factor for CKD-associated morbidity and may contribute to cardiovascular mortality in CKD patients [111] (Table 4).

Increased levels of interleukin-6 (IL-6) and tumor necrosis factor-alpha (TNF-α) involved in the inflammatory response predict poor outcome in patients with renal disease [112]. A proteomic approach identified an increased expression of serum IL-6 and TNF-α in the early stages of CKD, which highlights the importance of these inflammatory biomarkers for CKD patients’ diagnoses and management [113]. An observational study conducted by Kamińska et al. (2019), associated IL-6, but not TNF-α, to coronary artery calcification, a risk factor for cardiovascular mortality, in CKD and ESRD patients [114].

The overexpression of other pro-inflammatory cytokines, such as interleukin-8 (IL-8) and interleukin-18 (IL-18), has also been linked to renal function decline [113,115,116]. Likewise, soluble TNF receptors 1 and 2 (sTNFR1 and sTNFR2) showed an important role in the progression of atherosclerosis and kidney diseases [117,118].

Pentraxin 3 (PTX3) is produced by resident and innate immunity cells in peripheral tissues and increases rapidly in response to primary local activation of inflammation [119,120]. A cross-sectional study comprising two community-based cohorts of elderly subjects without CKD found that increased levels of PTX3 were associated with decreased eGFR and could independently predict incident CKD [121]. In ESRD patients undergoing dialysis, plasma PTX3 levels were shown to be more accurate than C-reactive protein (CRP), a traditional inflammatory marker, in predicting all-cause mortality. Moreover, plasma PTX3 levels were associated with other inflammatory markers (IL-6 and TNF-α), with adipokines disturbances and with the worst lipid risk profile [122]. This recent study highlights the association of PTX3 with multiple risk factors, such as inflammation and malnutrition. Furthermore, PTX3 was postulated to be a predictive marker of mortality in patients with advanced CKD [122,123].

Growth differentiation factor-15 (GDF-15) is a member of the transforming growth factor beta cytokine superfamily, and its expression may be induced in response to ischemia. GDF-15 was significantly associated with increased risk of CKD progression in several studies [124,125,126,127]. Nair et al. (2017) showed that circulating GDF-15 levels were strongly correlated with intrarenal expression of GDF-15, stating that GDF-15 may be a marker for intrarenal signaling pathways associated with CKD development and progression [124]. The Framingham study found associations between increased GDF-15 levels and the development of incident CKD with rapid decline of kidney function [128]. This study was limited to participants without CKD and suggests that increases in GDF-15 may help to identify higher risk for CKD development. Tuegel et al. (2018) found that elevated serum levels of GFD-15 were associated with an increased rate of heart failure in adults with CKD, but had no associations with atherosclerotic CVD events [126].

Inflammation and oxidative stress may contribute to DNA damage. In fact, DNA damage has been correlated with enhanced inflammatory states in different conditions, including CKD [129]. The quantification of circulating cell-free DNA (cfDNA), a measure of DNA damage, was proven to be helpful in detecting tumors in patients with renal cell carcinoma [130], and in predicting AKI after cardiac surgery [131].

Patients with CKD present different types of DNA damage and increased circulating levels of cfDNA [129]. Coimbra et al. (2017) analyzed the degree of genomic damage in ESRD patients undergoing hemodialysis (HD), and found that patients presented higher levels of CRP and cfDNA, correlating the persistent inflammatory state of those patients with the degree of DNA injury [132]. Furthermore, in non-dialyzed patients, urinary levels of cfDNA were predictors of unfavorable renal outcomes [133]. These findings were consistent with the results of Li et al. (2020), which demonstrated that increased serum cfDNA levels were associated with an increased risk of diabetic nephropathy in a cohort of diabetic patients [134], suggesting the potential of cfDNA as a biomarker of disease progression.

Watson et al. (2019) developed a Kidney Injury Test (KIT) based on the composite measurement and validation of six biomarkers across a set of 397 urine samples from patients with CKD, or with an increased risk for CKD (diabetic or hypertensive patients). The KIT analyzed DNA, protein, and metabolite markers, including cfDNA. In patients with normal renal function (eGFR) ≥ 90 mL/min/1.73 m^2^), the KIT score clearly identified those with predisposing risk factors for CKD, which could not be detected by eGFR or proteinuria [135].

### 2.5. Metabolomic Studies on CKD Biomarkers

In patients with CKD, metabolomic profiling identified several metabolites associated with alterations in carbohydrates, amino acids, nucleotides, and lipids metabolism. Since the progression of CKD is concomitant with metabolism alterations, these metabolites are being studied as potential biomarkers (Table 5).

The identification of these small molecules could help in assessing the multiple pathophysiological changes in CKD, since they might reflect early impairments in specific pathways. Lee et al. (2016) demonstrated that the presence of diabetes in CKD patients induces metabolic changes that are reflected in the levels of serum metabolites, such as arginine, *N*-acetyl-glycoprotein, glutamine, alanine and leucine [139]. CKD patients with diabetes present more severe metabolic changes than those without diabetes. However, these effects are particularly enhanced in the early stages rather than in advanced CKD [139]. A prospective study that enrolled 193 patients who developed CKD during the follow-up period and 193 matched controls found that decreased urinary levels of glycine and histidine were associated with incident CKD [140]. Additionally, Guo et al. (2019) identified 5-methoxytryptophan, canavaninosuccinate, acetylcarnitine, tiglylcarnitine and taurine as potential predictors of early CKD in a study population of 587 adults at all stages of CKD and of 116 healthy controls [141].

Metabolomic approaches identified that kynurenine, a metabolite of the amino acid L-tryptophan and an intermediate in the formation of NAD+, was associated with decreased eGFR and that alterations in the kynurenine/tryptophan ratio were associated with the presence of renal disease [142]. Additionally, kynurenine increases along with CKD progression, since patients with higher CKD stages exhibit higher kynurenine levels [143]. Debnath et al. (2017) showed that kynurenine was positively associated with TNF-α, suggesting that increased kynurenine might result from the ongoing inflammatory process [144].

Moreover, Ma et al. (2019) demonstrated that abnormalities in triacylglycerols and cardiolipins–phosphatidylethanolamines could discriminate CKD progression, preceding ESRD by several years [145]. Furthermore, Hu et al. (2018) identified three serum metabolites (fumarate, allantoin, and ribonate) associated with all-cause mortality from 299 CKD patients from the Modification of Diet in Renal Disease study, and 963 patients with CKD from the African American Study of Kidney Disease and Hypertension cohort [146].

Other potential metabolites related to kidney damage and/or GFR decline include citrulline [142,147], 1,5-anhydroglucitol [148,149], metabolites of the citric oxide pathway [150], and several others reviewed by Hocher and Adamski (2017) [151] and Cañadas-Garr et al. (2019) [152].

Besides metabolomic studies, there are other omic studies, namely proteomics, that are useful for the study of CKD. A comparison of CKD patients’ proteomic urinary profiles against those of healthy controls demonstrated that using a panel of 273 CKD-specific peptides could discriminate between the two groups [153]. The urinary peptide-based classifier CKD273 has been validated in different longitudinal and cross-sectional studies [154] and has sufficient sensitivity and specificity to allow differential diagnosis of CKD different etiologies [153]. The CKD273 represents one of most notable advances from proteomic studies of CKD biomarkers. The CKD273 is now commercially available as a non-invasive diagnostic test, allowing not only an earlier detection of CKD, but also patient stratification. Accordingly, in a cohort of patients with T2D and normoalbuminuria, the CKD273 proteomic biomarker panel was able to predict early diabetic nephropathy [155].

## 3. Future Perspectives and Conclusions

There is an emerging need for the identification of reliable early biomarkers of kidney injury, of progression of the disease and of morbidity and mortality risk. The reliance on only traditional biomarkers may result in a long time lapse, along which successful interventions could be applied. Most of these potentially new biomarkers identified from experimental studies may detect renal injury earlier than traditional biomarkers. Moreover, newer biomarkers can provide information about the pathophysiological mechanism (s) underlying renal disease, predict disease progression, severity and associated cardiovascular and/or all-cause mortality.

Creatinine limitations have been well known for more than thirty years [168]. However, creatinine is still the standard biomarker used for CKD evaluation in clinical practice. Newer glomerular filtration markers, such as BTP and B2M, have proven their potential to improve the accuracy and the predictive value of GFR estimation. With the increased use of cystatin C, BTP and B2M, GFR estimation is likely to undergo further improvements [18,26]. Other markers, such as NGAL, KIM-1 and L-FABP might be helpful in identifying early tubular damage, especially in the creatinine-blind range, and before pathological and irreversible changes occur. Moreover, their specificity allows the recognition of kidney damage separately from changes in kidney function. The initiation and the progression of endothelial dysfunction, closely related to the inflammatory response, is simultaneously a cause and a consequence of both CKD and CVD [110], which highlights the importance of early detection of such pathological pathways through early endothelial dysfunction and/or inflammatory markers assessment.

Advancing laboratory techniques that allow the concomitant analysis of multiple biomarkers are currently being used and have largely impacted the discovery of new biomarkers. Dozens of dysregulated peptides and metabolites were identified by proteomic and metabolomic studies. Furthermore, microRNA analysis studies have highlighted its implication in the pathogenesis of CKD [169,170,171], particularly in kidney fibrotic transformation [172]. Different microRNA expression profiles were identified under different renal disease conditions [173]. Due to microRNAs functional promiscuity, since they usually target proteins from various pathways, confirmation studies are still required to clarify its role in the clinical diagnostic setting. Despite their promising potential, metabolite profiling technologies and microRNA analysis are still expensive and time consuming, explaining why their application is not widely available.

It is unlikely that a single biomarker can predict CKD progression and identify the multiple pathophysiological processes involved in CKD progression or the underlying primary renal disease. Instead, a panel measuring multiple biomarkers seems to be a more reasonable approach to better predict CKD development and to access the outcomes of the disease, since there are several different mechanisms by which CKD can result. Additionally, the incorporation of multiple serum and/or urinary biomarkers is likely to be synergetic in predicting renal failure while decreasing the impact of non-renal determinants for each biomarker alone [174]. Panels of biomarkers for earlier detection of CKD are expected to include biomarkers related to the primary disease cause, while biomarkers for predicting disease progression are more likely to be related to the sequentially reduced renal function. Although it seems reasonable, the availability of such tools depends on the characterization of patients’ profiles against healthy controls and the validation of methods for analytical procedures.

CKD burden is not limited to the disease itself, extending to ESRD- and CKD-related CVD and other complications. Identification of CKD at early stages allows more rapid interventions in order to control disease progression and management of risk factors. This will translate in better patients’ prognoses and economic benefits related to decreased healthcare expenses.

In summary, further validation for most of these new potential biomarkers requires larger studies with standardized methodologies in order to be implemented in routine CKD management, either for an early diagnosis or for the detection of disease worsening. Moreover, the search for panel (s) of biomarkers that can synergistically detect renal disease or a poor outcome for renal patients is important.

## Figures and Tables

**Table 1:** Research studies on biomarkers of renal function and main outcomes in CKD patients.

Year	Study Type	Study Population	Biomarker (s)	Study Outcomes	Reference
2015	Prospective cohort	9703 participants from the ARIC	serum B2M	Greater than 30% decline in B2M may be less common, but appears to be more specific for ESRD than equivalent changes in eGFR based on serum creatinine	[24]
2015	Prospective cohort	250 Pima Indians with T2D	serum BTP, B2M	BTP and to a lesser extent B2M were associated with ESRD; only higher serum concentrations of B2M were associated with increased mortality risk in this population	[22]
2015	Cross-sectional	93 CKD patients at stages 1–5	urinary klotho	Decreased tubular phosphate reabsorption was associated with decreased eGFR, but it was not associated with urinary klotho levels	[39]
2016	Cross-sectional	355 CKD patients, classified in the different stages of CKD	urinary BTP	BTP is present in the urine of patients with normal GFR, and its urinary excretion progressively increases along with the reduction of GFR; clearance of BTP progressively increases with the reduction of GFR	[16]
2016	Cross-sectional	109 CKD patients with T2D and 32 healthy controls	serum klotho	Serum klotho levels were significantly elevated in diabetic patients; klotho levels decreased with increasing albumin excretion	[33]
2016	Retrospective cohort	3551 participants with CKD from MDRD, AASK and CRIC studies	serum BTP, B2M	BTP and B2M are less influenced by age, sex and race than creatinine and less influenced by race than cystatin C, but provide less accurate GFR estimates	[10]
2016	Prospective cohort	3613 adults from the CRIC study	serum BTP, B2M	BTP and B2M were independent predictors of ESRD and all-cause mortality, but only B2M was an independent predictor cardiovascular events	[40]
2017	Prospective cohort	2496 participants from the Health Aging and Body Composition study	serum klotho	Higher klotho levels were associated with lower odds of kidney function decline, but not with incident CKD	[41]
2017	Cross-sectional	50 individuals with type 2 diabetes and 25 healthy controls	serum BTP	BTP level was significantly higher in T2Dwith the microalbuminuria group than T2DM with normoalbuminuria and control groups	[18]
2017	Cross-sectional	566 individuals aged 70+ from the Berlin Initiative Study	serum BTP	Combination of creatinine, cystatin C and BTP showed the best prediction of GFR; single usage of BTP showed the worst prediction within models with only one biomarker	[42]
2017	Prospective cohort	317 participants from MDRD and 373 from AASK	serum BTP, B2M	Declines in eGFR based on the average of four filtration markers (creatinine, cystatin C, BTP, and B2M) were consistently associated with progression to ESRD; only the decline in eGFR-BTP was significantly more strongly associated with ESRD risk	[23]
2017	Meta-analysis	23,318 individuals from six different studies	serum BTP, B2M	eGFR-BTP, eGFR-B2M, and their average showed stronger risk associations with ESRD and all-cause mortality when compared with eGFRcr	[27]
2017	Cross-sectional	Elderly participants from the AGES-Kidney study (683) and the MESA-Kidney (273)	serum BTP, B2M	eGFR-cys, eGFR-B2M and eGFR-BTP had significantly less strong residual associations with age and sex than eGFRcr	[11]
2018	Cross-sectional	125 maintenance hemodialysis patients	serum klotho	Klotho levels were associated with the degree of bone mineral density; osteoporosis groups presented lower levels than the normal bone mass group	[37]
2018	Prospective cohort	112 adults with stages 1–5 CKD	serum klotho	Klotho levels were positively associated with baseline eGFR; reduction in klotho levels was associated with renal function decline	[31]
2018	Cross-sectional	150 patients with CKD at stages 1–4 and 50 healthy controls	serum BTP	Increased BTP concentrations in CKD patients are highly significantly correlated with the concentrations of Cr and Cys; BTP had a higher value of correlation with mGFR	[17]
2018	Systematic review and meta-analysis	9 publications, comprising 1457 CKD patients	serum klotho	There was a positive correlation between serum klotho levels and eGFR; no significant correlations were found between serum klotho levels and calcium and phosphorus circulating levels	[32]
2018	Cross-sectional	566 individuals aged 70+ from the Berlin Initiative Study	serum BTP	The addition of BTP to serum creatinine-based eGFR equations does not result in the same improvement as the addition of Cys	[25]
2018	Cross-sectional	50 healthy term neonates	serum BTP	BTP concentrations were positively associated with the concentrations of serum Cr level; inverse serum BTP is associated with estimated GFR level among neonates	[43]
2019	Prospective cohort	86 adults with stable CKD	serum BTP, B2M	The addition of BTP/B2M eGFR to Cr/cysC eGFR equations did not improve GFR estimation	[26]
2019	Systematic Review and Meta-analysis	8 cohort studies with 3586 participants	serum klotho	Klotho levels were positively correlated with the eGFR; lower klotho levels were significantly associated with an increased risk of poor kidney outcomes	[44]
2019	Prospective cohort	107 diabetic patients with CKD at stages 2 and 3	serum klotho	Lower levels of klotho were associated with cardiac pathological changes and higher CVD risk	[34]
2019	Prospective cohort	79 CKD patients on hemodialysis	serum klotho	Lower klotho levels were associated with the risk of CVD, independently from factors associated with mineral bone disease	[35]
2019	Cross-sectional	286 CKD patients at stages 2–5	serum klotho	The serum levels of inflammatory markers were negatively associated with klotho levels	[36]
2019	Cross-sectional	152 patients with CKD at stages 3–5 and 30 healthy controls	serum klotho	eGFR reduction was associated with decreased klotho levels; serum phosphate levels were negatively associated with klotho levels	[38]
2020	Cross-sectional	1066 participants with Cr and Cys and 666 with all 4 markers	serum BTP, B2M	eGFR-B2M and eGFR-BTP were not more accurate than eGFR-cr and eGFR-cys; accuracy was significantly better for the eGFR equation considering the four markers when compared to eGFRcr-cys equation	[15]
2020	Prospective cohort	830 Chinese CKD patients	serum B2M	The B2M equation had smaller bias in the subgroup of GFR 60–89 mL/min/1.73 m^2^, but a larger bias and worse precision and accuracy in the subgroup of GFR > 90 mL/min/1.73 m^2^ when compared to the CKD-EPI equation	[45]
2020	Cross-sectional	1793 patients from the KNOW-CKD study	serum klotho	Decreased klotho levels correlated negatively with phosphate levels and with the degree of proteinuria	[46]

Abbreviations: AASK, African American Study of Kidney Disease and Hypertension; AGES, Age, Gene/Environment Susceptibility; ARIC, Atherosclerosis Risk in Communities; B2M, beta-2 microglobulin; BTP, beta trace protein; CKD, chronic kidney disease; CKD-EPI, Chronic Kidney Disease Epidemiology Collaboration; Cr, creatinine; CRIC, Chronic Renal Insufficiency Cohort; CVD, Cardiovascular disease; Cys, cystatin C; eGFR, estimated glomerular filtration rate; ESRD, End-stage renal disease MDRD, Modification of Diet in Renal Disease; MESA, Multi-Ethnic Study of Atherosclerosis; mGFR, measured glomerular filtration rate; T2D, type 2 diabetes.

**Table 2:** Research studies on biomarkers of renal tubular lesions and main outcomes in CKD patients.

Year	Study Type	Study Population	Biomarker (s)	Study Outcomes	Reference
2015	Prospective cohort	1245 women aged ≥ 70 from the general population	plasma NGAL	NGAL is of modest clinical utility in predicting renal function decline and acute or chronic renal disease-related events in individuals with mild-to-moderate CKD	[71]
2015	Cross-sectional	276 type 2 diabetic patients with CKD at stage 1 and 2	urinary L-FABP	Urinary L-FABP was significantly correlated with UACR in the early stages of CKD	[66]
2015	Prospective cohort	138 patients with CHF	urinary KIM-1, NGAL, NAG	In patients with CKD progression, KIM-1 and NAG were elevated in contrast to NGAL; KIM-1 and NAG were negatively correlated with ejection fraction and eGFR	[58]
2016	Cross-sectional	355 patients with CKD at stages 1–5 and 71 patients without CKD	plasma and urinary UMOD	UMOD allowed the identification of patients without CKD and patients at any stage of CKD; Plasma UMOD appears to outperform urinary uromodulin as a CKD marker	[69]
2016	Prospective cohort	244 adult patients with CKD	urinary NAG, L-FABP	Elevated urinary L-FABP and low eGFR were associated with the development of ESRD and CVD, irrespective of diabetes	[65]
2016	Cross-sectional	170 patients with CKD at stages 1 to 5 and 30 healthy individuals	serum UMOD	Serum UMOD concentrations in CKD patients were lower than in healthy subjects, and the lower concentrations were associated with more advanced stages	[68]
2017	Case–control	74 adult CKD patients (stages 3–5) and 25 healthy subjects	urinary L-FABP	L-FABP shows a negative correlation with GFR and a positive correlation with UAC; in patients without albuminuria, L-FABP was associated with renal function decline	[64]
2017	Prospective cohort	250 patients with CKD at stages 1–5, including 111 on hemodialysis	urinary KIM-1, NGAL, NAG	NGAL was moderately correlated with the 5 stages of CKD, while KIM-1 and NAG were also correlated, but weakly	[59]
2018	Cross-sectional	80 patients with T2D without significant decrease in eGFR and albuminuria	urinary NGAL, KIM-1, UMOD	Urinary NGAL and KIM-1 correlated positively with albuminuria; all markers differed significantly between patients with moderately increased albuminuria compared to those with normal to mildly increased albuminuria	[55]
2018	Prospective cohort	2813 patients from C-STRIDE study	serum UMOD	Higher incidence rates of ESRD, CVD and death were associated with decreased UMOD levels	[72]
2018	Cross-sectional	132 patients with CKD at stages 1–5 and 33 patients without CKD	serum UMOD	UMOD levels inversely correlated with creatinine and creatinine/cystatin C-based eGFR	[70]
2018	Case–control	324 participants from the SPRINT trial (162 who developed CKD during the follow-up and 162 matched controls)	urinary KIM-1, NGAL, UMOD	Only baseline concentrations of KIM-1 were associated with the development of incident CKD during the follow-up	[54]
2018	Prospective cohort	527 adults with type 1 diabetes from the CACTI study	plasma KIM-1, NGAL, UMOD	NGAL and UMOD were associated with UACR and incident impaired GFR over the 12-year follow-up period	[56]
2018	Prospective cohort	143 patients with stable CKD with diverse etiologies	urinary NGAL, KIM-1	Neither NGAL nor KIM-1 provided robust prognostic information on the loss or renal function in a heterogeneous CKD population	[73]
2018	Cross-sectional	109 biopsy-proven lupus nephritis patients and 50 healthy individuals	urinary NGAL, KIM-1	Patients with active lupus nephritis exhibited elevated urinary levels of KIM-1 and NGAL compared with patients in remission and controls; KIM-1 levels correlated with tubular atrophy and interstitial inflammatory lesions	[74]
2019	Prospective cohort	230 CKD patients stages 1 to 5	urinary UMOD	UMOD concentrations were positively associated with eGFR and inversely associated with proteinuria; UMOD levels were independently associated with ESRD or rapid loss of eGFR	[75]
2019	Prospective cohort	933 individuals aged ≥65 years from the CHS study	serum UMOD	Lower UMOD was associated with the development of ESRD independently of eGFR, UACR, and cardiovascular and CKD risk factors	[76]
2019	Cross-sectional	39 children with kidney cysts, including 20 subjects with ADPKD, and 20 controls	urinary and serum L-FABP	Higher concentration of L-FABP in serum and urine indicated early damage to the renal parenchyma, detectable before the onset of hypertension and other organ damage	[77]
2019	Cross-sectional	165 biopsy-proven CKD patients and 64 healthy controls	urinary NAG, KIM-1, NGAL	All biomarkers were significantly increased in patients, but their values were similar for patients with moderate and severe tubular injury	[78]
2019	Systematic review and meta-analysis	22 studies involving 683 healthy individuals and 3249 diabetic patients	serum and urinary NGAL	Both urinary and serum NGAL showed an increasing trend, in parallel with albuminuria and progression of the disease, estimated by eGFR; the highest concentrations were achieved in patients with the highest severity of diabetic nephropathy	[52]
2019	Cross-sectional	209 T2D normoalbuminuric patients with or without CKD	urinary NGAL	Levels of urinary NGAL were elevated in patients with renal insufficiency and negatively related to eGFR in T2D patients with normoalbuminuria	[79]
2019	Prospective cohort	2377 participants from SPRINT trial with non-diabetic CKD	urinary UMOD	Lower uromodulin levels were associated with higher risk of CVD events and mortality, independently of eGFR, UACR, and other risk factors	[80]
2019	Cross-sectional	287 T2D patients and 42 healthy controls	urinary NGAL	Urinary NGAL was significantly correlated with the UACR in patients with T2D	[81]
2020	Cross-sectional	133 patients with diabetes and 39 healthy controls	urinary NGAL	Patients with severely increased albuminuria had higher levels of NGAL compared to patients with normal albuminuria and controls	[51]
2020	Prospective cohort	4739 participants of the population-based Malmö Diet and Cancer Study	plasma KIM-1	Plasma KIM-1 was able to predict the future decline of eGFR and the risk of CKD in healthy participants	[82]

Abbreviations: ADPKD, autosomal dominant polycystic kidney disease; CACTI, Coronary Artery Calcification in Type 1 Diabetes study; CHF, congestive heart failure; CHS, Cardiovascular Health Study; CKD, chronic kidney disease; C-STRIDE, Chinese Cohort Study of Chronic Kidney Disease; CVD, cardiovascular disease; eGFR, estimated glomerular filtration rate; ESRD, end-stage renal disease; KIM-1, kidney injury molecule-1; L-FABP, liver-type fatty acid binding protein; NAG, N-acetyl-β-glucosaminidase; NGAL, neutrophil gelatinase-associated lipocalin; SPRINT, Systolic Blood Pressure Intervention Trial; T2D, type 2 diabetes; UACR, urine albumin-to-creatinine ratio; UMOD, uromodulin.

**Table 3:** Research studies on biomarkers of endothelial dysfunction and main outcomes in CKD patients.

Year	Study Type	Study Population	Biomarker (s)	Study Outcomes	Reference
2015	Cross-sectional	201 patients with CKD and 201 controls	plasma ADMA	Plasma ADMA levels were associated with CKD severity measured by eGFR and/or albuminuria	[104]
2016	Prospective cohort	259 patients with CKD at stages 1–5	serum ADMA	Patients with ADMA levels above the median value had an increased risk of all-cause mortality and CVE	[105]
2016	Cross-sectional	35 pre-dialysis CKD patients, 40 on hemodialysis, and 15 healthy subjects	plasma ADMA	Plasma ADMA concentration was associated with disadvantageous changes in left ventricular structure and function	[93]
2016	Prospective cohort	463 individuals with CKD at stages 3–5 from the CRISIS Study	plasma fetuin-A	There was no clear association between fetuin-A and risk for RRT, CVE, and death	[106]
2018	Prospective cohort	528 adult CKD patients at stages 2–4	plasma ADMA	eGFR was inversely correlated with plasma levels of ADMA	[107]
2018	Systematic review and meta-analysis	6 articles, involving 616 CKD patients	plasma ADMA	Levels of circulating ADMA were positively related to CIMT in CKD patients	[92]
2018	Prospective cohort	162 hypertensive CKD patients, free from albuminuria	plasma ADMA	High ADMA levels were associated with the progression of albuminuria in hypertensive patients, with and without type 2 diabetes	[85]
2019	Cross-sectional	651 elderly subjects from KSHAP cohort study	plasma ADMA	eGFR levels were inversely associated with ADMA concentrations	[91]
2019	Cross-sectional	176 CKD patients and 64 control subjects	plasma ADMA	Plasma ADMA levels were similar in the control group and stage 1 CKD patients; in other stages, ADMA levels were significantly higher in comparison to the control subjects	[84]
2019	Systematic review and meta-analysis	13 studies comprising 5169 CKD patients	serum fetuin-A	CKD patients with the lowest fetuin-A levels had a 92% greater risk of all-cause mortality compared with those with the highest levels	[98]
2019	Cross-sectional	238 CKD patients (stages 3–5)	serum fetuin-A	Fetuin-A levels in ESRD patients were significantly lower than those from patients at stages 3 and 4 CKD; fetuin-A was negatively correlated with vascular calcification score and CIMT	[97]
2020	Cross-sectional	43 adult patients with CKD and 43 healthy controls	plasma ADMA	Levels of ADMA positively correlate with CKD severity; FMD was significantly decreased in CKD patients, and negatively correlated with ADMA levels	[86]

Abbreviations: ADMA, asymmetric dimethylarginine; CIMT, carotid intima-media thickness; CKD, chronic kidney disease; CRISIS, Chronic Renal Insufficiency Standards Implementation Study; CVE, cardiovascular event; eGFR, estimated glomerular filtration rate; ESRD, end-stage renal disease; FMD, flow-mediated dilation; RRT, renal replacement therapy; T2D, type 2 diabetes.

**Table 4:** Research studies on biomarkers of inflammation and main outcomes in CKD patients.

Year	Study Type	Study Population	Biomarker (s)	Study Outcomes	Reference
2016	Prospective cohort	746 individuals with GFR > 60mL/min/1.73 m^2^	serum PTX3	Higher PTX3 levels are associated with lower GFR and independently predict incident CKD in the elderly	[121]
2016	Prospective cohort	3430 patients with reduced eGFR from the CRIC study	plasma IL-6, TNF-α	Elevated plasma levels of TNF-α were associated with rapid loss of kidney function in CKD patients	[108]
2016	Prospective cohort	543 patients with stage 5 CKD	plasma IL-6, TNF- α	IL-6 and TNF-a could predict all-cause mortality risk; only IL-6 could classify clinical CVD	[112]
2017	Prospective cohort	521 adults with CKD from the C-PROBE and the SKS studies	plasma or serum GDF-15	Circulating GDF-15 levels were strongly correlated with intrarenal expression of GDF15 and significantly associated with increased risk of CKD progression	[124]
2017	Prospective cohort	984 CKD patients stages 1–5	serum TNFR1, TNFR2	TNFR1 and 2 were associated with CVD and other risk factors in CKD, independently of eGFR	[117]
2017	Cross-sectional	1816 community residents randomly selected from the Dong-gu study	plasma PTX3	A significantly higher risk of CKD was found in the group with the highest plasma levels of PTX3 when compared to the group with the lowest levels	[136]
2017	Prospective cohort	78 stage 5 CKD patients (51 on hemodialysis and 27 on pre-dialysis)	serum PTX3, IL-6, CRP	In contrast to CRP levels, baseline PTX3 levels predicted CV mortality independently of classic CV risk factors; PTX3 levels also significantly predicted mortality	[123]
2017	Prospective cohort	39 ESRD patients under HD and 15 healthy controls	serum cfDNA	ESRD patients had a significantly higher value when compared to controls; cfDNA correlated positively with CRP levels in ESRD patients	[132]
2018	Prospective cohort	883 adults at any stage CKD from the SKS or the C-PROBE studies	serum GDF-15	Adults with CKD and higher circulating levels of GDF-15 presented greater mortality; elevated GDF-15 was also associated with an increased rate of HF	[126]
2018	Prospective cohort	200 patients with T2D	plasma GDF-15	Higher GDF-15 improved risk prediction of decline in kidney function; in patients with T2D and microalbuminuria, higher GDF-15 was independently associated with all-cause mortality	[137]
2018	Cross-sectional	201 patients with CKD and 201 controls	plasma PTX3	Plasma PTX3 levels were increased in patients with CKD when compared to controls	[138]
2019	Prospective cohort	3664 participants with CKD from the CRIC study	plasma GDF-15	GDF-15 was significantly associated with an increased risk of CKD progression	[125]
2019	Prospective cohort	57 CKD patients at stages 3–5 and 19 healthy controls	serum IL-6, TNF- α	TNF and IL-6 were significantly higher in more advanced CKD stages; IL-6, but not TNF- α, was associated with 5-year risk of all-cause mortality in CKD patients	[114]
2019	Prospective cohort	318 ESRD patients, undergoing HD and 22 healthy controls	plasma PTX3, IL-6, TNF- α, CRP	When comparing inflammatory mediators, the increase in PTX3 levels was the only predictor of all-cause mortality in dialysis patients	[122]
2019	Prospective cohort	124 patients with CKD (stages 1–5)	plasma and urinary cfDNA	No correlations were found between cfDNA levels and CKD staging; higher urinary levels of cfDNA were associated with worse renal outcomes at 6 months	[133]
2020	Cross-sectional	219 adult CKD patients (stages 2–5) from the GCKD study	plasma GDF-15	GDF-15 was significantly elevated in CKD patients and showed a significant inverse correlation with eGFR	[127]
2020	Cross-sectional	117 T2D patients and 11 healthy controls	serum and urinary IL-8, IL-18	Serum and urinary levels of IL-8 and IL-18 were positively correlated with podocyte damage, peritubular dysfunction, and albuminuria, and negatively correlated with eGFR	[115]
2020	Prospective cohort	2428 SPRINT participants with CKD	urinary IL-18	Urinary IL-18 was associated with eGFR decline and may help to detect subtle changes in eGFR	[116]
2020	Prospective cohort	160 patients with DN	serum cfDNA	Serum cfDNA levels were significantly negatively associated with the eGFR changes during the follow-up	[134]

Abbreviations: CKD, chronic kidney disease; C-PROBE, Clinical Phenotyping and Resource Biobank Study; CRIC, Chronic Renal Insufficiency Cohort; CRP, C-reactive protein; CVD, cardiovascular disease; DN, diabetic nephropathy; eGFR, estimated glomerular filtration rate; GCKD, German Chronic Kidney Disease; GDF-15, growth differentiation factor-15; HD, hemodialysis; HF, heart failure; IL-6, interleukin-6; IL-8, interleukin-8; IL-18, interleukin-18; PTX3, pentraxin-3; SKS, Seattle Kidney Study; SPRINT, Systolic Blood Pressure Intervention Trial; T2D, type 2 diabetes; TNF-α, tumor necrosis factor alfa; TNFR, tumor necrosis factor receptor.

**Table 5:** Research studies on metabolomics and main outcomes, in CKD patients.

Year	Study Type	Study Population	Study Outcomes	Reference
2016	Cross-sectional	27 patients with CKD at stages 3–5	Kidney function decline was associated with an increase in the inflammation marker neopterin and the metabolism of tryptophan via the kynurenine pathway	[156]
2016	Prospective cohort	118 patients with CKD at stages 3–5	Sixteen metabolites, from variable metabolic pathways, were related to higher risk of kidney function deterioration in advanced CKD patients	[157]
2016	Case–control	200 patients with rapid renal disease progression and 200 stable controls	Ten metabolites were associated with CKD progression; six (uric acid, glucuronate, 4-hydroxymandelate, 3-methyladipate/pimelate, cytosine, and homogentisate) were higher in cases than controls, whereas four (threonine, methionine, phenylalanine, and arginine) were lower	[158]
2016	Prospective cohort	1735 participants in the KORA F4 study	Six metabolites (N-acetylalanine, N-acetylcarnosine, C-mannosyltryptophan, erythronate, pseudouridine, and O-sulfo-L-tyrosine) were associated with eGFR and CKD in both studies and showed high correlation with established kidney function markers	[159]
		1164 individuals in the TwinsUK registry		
2016	Cross-sectional	291 pre-dialysis CKD patients and 56 healthy controls	The presence of diabetes affects the metabolic phenotypes of CKD patients at an early stage, and those differences are attenuated with CKD progression	[139]
2017	Case–control	193 patients with incident CKD from the Framingham Study and 193 matched controls	Lower urinary levels of glycine and histidine were associated with a higher risk of incident CKD; moreover, the authors identified several novel associations with urinary metabolites and genetic variations	[140]
2017	Cross-sectional	60 T2D patients with all stages of CKD from the FIND study	Tryptophan levels were inversely correlated with CKD staging, while its metabolites were positively associated with the severity of kidney disease; kynurenine was positively correlated with TNF-a levels	[144]
2017	Cross-sectional	589 CKD patients from the MDRD study	Five metabolite associations (kynurenate, homovanillate sulfate, hippurate, N2,N2-dimethylguanosine, and 16-hydroxypalmitate) showed consistently higher levels in ADPKD compared with glomerular disease and CKD of other causes	[147]
2017	Cross-sectional	22 non-diabetic CKD stage 3–4 patients and 10 healthy controls	Urinary levels of 27 metabolites and plasma concentration of 33 metabolites differed significantly in CKD patients compared to controls; the citric acid cycle pathway was the most affected, with reduced urinary excretion of citrate, cis-aconitate, isocitrate, 2-oxoglutarate and succinate	[150]
2017	Cross-sectional	20 CKD patients at stage 3 and 20 at stage 5, and 20 healthy controls	Glycoursodeoxycholic acid and 2-hydroxyethane sulfonate were downregulated in the urine of patients, and pregnenolone sulfate was also found to be decreased in plasma when compared to controls	[160]
2018	Prospective cohort	56 Brazilian macroalbuminuric CKD patients	Lower levels of 1,5-AG, norvaline and l-aspartic acid were significantly associated with the risk of a combined outcome of mortality, dialysis need or creatinine doubling	[148]
2018	Retrospective cohort	227 patients with CKD and a nested subgroup of 57 for follow up	Eleven metabolites from various metabolic pathways were associated with reduced eGFR; increased urinary concentrations of betaine and myo-inositol were found to be prognostic markers of CKD progression	[161]
2018	Prospective cohort	299 CKD patients from the MDRD study and 963 from the AASK cohort	Serum metabolites fumarate, allantoin, and ribonate were associated with a higher risk of mortality in two cohorts of patients with CKD	[146]
2018	Prospective cohort	1765 Chinese adults with eGFR ≥ 60 mL/min per 1.73 m2	Elevated plasma levels of cysteine and several acylcarnitines were associated with eGFR reduction, independent of baseline eGFR and other conventional risk factors	[162]
2019	Cross-sectional	587 adults with all stages of CKD and 116 healthy controls	Five serum metabolites (5-MTP, canavaninosuccinate, acetylcarnitine, tiglylcarnitine and taurine) were identified to estimate kidney filtration and enhance earlier CKD prediction	[141]
2019	Prospective cohort	1582 participants from the AASK and MDRD studies	The serum metabolites 4-hydroxychlorthalonil and 1,5-AG and the phosphatidylethanolamine metabolic pathway were strongly associated with proteinuria in CKD	[149]
2019	Cross-sectional	30 patients with CKD at stages 3 and 4 and 30 healthy volunteers	More significant changes in acylcarnitines, carbohydrates (such as glucose and myo-inositol), and glycerophospholipid metabolism pathways were found in CKD patients than in controls	[163]
2019	Retrospective cohort	214 CKD patients from the CPROBE and 200 from the CRIC studies	In CKD patients, changes in the triacylglycerols and cardiolipins-phosphatidylethanolamines preceded the clinical outcomes of ESRD by several years	[145]
2019	Cross-sectional	1243 participants from the BHS and 260 from the MESA studies	This study identified 39 novel metabolites in sub-pathways previously associated with kidney function, and 12 novel metabolites in sub-pathways with novel associations	[164]
2019	Retrospective cohort	454 patients with CKD at stages 3 and 4 from the Progredir Cohort Study	D-malic acid, acetohydroxamic acid, butanoic acid, ribose, glutamine, trans-aconitic acid, lactose and an unidentified molecule (m/z 273) were positively related to the risk of overall mortality, while docosahexaenoic acid was inversely related to this risk; lactose, 2-O-glycerol-α-d-galactopyranoside, and tyrosine were associated with ESRD progression	[165]
2019	Cross-sectional	140 CKD patients and 144 healthy subjects	CKD patients presented significantly lower serum levels of 3-indolepropionic acid and higher serum levels of indoxyl sulfate and p-cresol sulfate when compared to controls	[166]
2020	Prospective cohort	1741 subjects from the Ansan-Ansung population study	Researchers found 22 metabolites associated with eGFR and CKD prevalence; citrulline, kynurenine, and the kynurenine/tryptophan ratio were associated with incident CKD	[142]
2020	Prospective cohort	184 patients with CKD at stages 1–5 from the CPROBE study	Kynurenic acid, 3-hydroxykynurenine and kynurenine were increased with CKD stage progression; higher tryptophan levels at baseline were associated with lower odds of incident CVD	[143]
2020	Prospective cohort	501 patients with ADPKD, with different stages of CKD	Four urinary metabolites (myo-inositol, 3-hydroxyisovalerate, ADMA and creatinine) were strongly associated with baseline eGFR; the urinary alanine/citrate ratio showed the best association with eGFR decline	[167]

Abbreviations: 1,5-AG, 1,5-anhydroglucitol; 5-MTP, 5-methoxytryptophan; AASK, African American Study of Kidney Disease and Hypertension; ADMA, asymmetric dimethylarginine; ADPKD, autosomal dominant polycystic kidney disease; BHS, Bogalusa Heart Study; CKD, chronic kidney disease; CPROBE, Clinical Phenotyping Resource and Biobank Core; CRIC, Chronic Renal Insufficiency Cohort Study; CVD, cardiovascular disease; DKD, diabetic kidney disease; eGFR, estimated glomerular filtration rate; ESRD, end-stage renal disease; FIND, Family Investigation of Nephropathy in Diabetes; KORA, Cooperative Health Research in the region of Augsburg; MDRD, Modification of Diet in Renal Disease; MESA, Multi-Ethnic Study of Atherosclerosis; T2D, type 2 diabetes; TNF-α, tumor necrosis factor alph

## Abbreviations

CKD	Chronic kidney disease
ESRD	End-stage renal disease
CVD	Cardiovascular disease
GFR	Glomerular filtration rate
BTP	Beta trace protein
B2M	β2-microglobulin
T2D	Type 2 diabetes
eGFR	Estimated glomerular filtration rate
NGAL	Neutrophil gelatinase-associated lipocalin
KIM-1	Kidney injury molecule-1
NAG	N-acetyl-β-D-glucosaminidase
L-FABP	Liver-type fatty acid binding protein
UMOD	Uromodulin
T1D	Type 1 diabetes
AKI	Acute kidney injury
NO	Nitric oxide
ADMA	Asymmetric dimethylarginine
IL-6	Interleukin-6
TNF-α	Tumor necrosis factor-α
PTX3	Pentraxin 3
CRP	C-reactive protein
GDF-15	Growth differentiation factor-15
cf-DNA	Cell-free DNA
